# Absorbable Synthetic Meshes in Implant-Based Breast Reconstruction After Subcutaneous Mastectomy: A Systematic Review with Clinical and Economic Considerations

**DOI:** 10.3390/medicina62091682

**Published:** 2026-09-02

**Authors:** Aurimas Dobilinskas, Loreta Pilipaitytė

**Affiliations:** Department of Plastic and Reconstructive Surgery, Hospital of Lithuanian University of Health Sciences Kauno Klinikos, 50161 Kaunas, Lithuania; loreta.pilipaityte@kaunoklinikos.lt

**Keywords:** breast reconstruction, implant-based breast reconstruction, absorbable mesh, polyglactin, polydioxanone, prepectoral reconstruction, material costs

## Abstract

*Background and Objectives:* Implant-based breast reconstruction following subcutaneous mastectomy is widely performed; however, the optimal strategy for implant support remains controversial. Absorbable synthetic meshes have emerged as lower-cost alternatives to acellular dermal matrix (ADM), yet their clinical and economic value remains incompletely defined. *Materials and Methods:* A systematic review was conducted in accordance with PRISMA guidelines using PubMed, Scopus, and Web of Science databases. Studies evaluating implant-based breast reconstruction with absorbable synthetic meshes, ADM, or without mesh were included. Outcomes of interest comprised postoperative complications, operative time, material costs, aesthetic outcomes, and patient-reported satisfaction. Methodological quality and risk of bias were assessed using design-appropriate appraisal tools. *Results*: Absorbable synthetic meshes were associated with low complication rates, including infection (3–8%), seroma (0–5%), hematoma (1–4%), skin necrosis (4–6%), and implant loss (3–6%). Compared with ADM, complication rates appeared comparable, while shorter operative times were reported in comparative studies. Material cost reductions of up to 90% were reported in comparative studies. Several studies reported favorable aesthetic outcomes and high patient satisfaction, although assessment methods were not standardized. Most included studies were retrospective or single-arm observational studies, with substantial clinical and methodological heterogeneity and an overall moderate risk of bias. *Conclusions*: Available evidence, derived predominantly from studies of polyglactin mesh, suggests that absorbable synthetic meshes may represent a feasible option for selected implant-based breast reconstructions, with potential advantages in terms of material costs and operative time. Evidence regarding polydioxanone mesh remains very limited, and the methodological limitations of the available studies preclude definitive conclusions.

## 1. Introduction

Breast reconstruction with implants following subcutaneous mastectomy has become an integral component of modern oncologic and plastic and reconstructive surgery, significantly contributing to patients’ psychosocial well-being and overall quality of life. Over the past decade, implant-based breast reconstruction has emerged as the dominant approach in many centers worldwide, as it enables faster aesthetic outcomes, shorter operative times, and more rapid postoperative recovery compared with autologous microsurgical techniques [1,2]. Furthermore, patients undergoing implant-based reconstruction typically experience quicker return to daily activities and reduced perioperative burden.

Traditionally, implant-based breast reconstruction following subcutaneous mastectomy has most commonly been performed in the subpectoral plane. While this approach effectively restores breast volume and contour, it is associated with several clinically relevant drawbacks. Patients often experience postoperative pain, restricted upper limb mobility for several months, delayed rehabilitation, and functional limitations [3]. Additionally, during the early postoperative period, before the formation of a stabilizing fibrous capsule, the implant may lack adequate support within the created pocket. This may result in implant rotation or displacement, leading to suboptimal positioning.

Moreover, as the pectoralis major muscle adheres to the developing capsule, muscle contraction can cause implant displacement, resulting in breast animation deformity. This phenomenon represents a significant functional and aesthetic concern, particularly in physically active patients.

To overcome these limitations and reduce both functional impairment and aesthetic complications, the use of synthetic meshes has been increasingly adopted. Both absorbable and non-absorbable meshes allow for implant placement in a prepectoral (subcutaneous) position. These materials provide structural support, help maintain implant position—particularly in the lower pole of the breast—and may reduce complications such as seroma formation, skin necrosis, and implant malposition in appropriately selected patients [3].

Various adjunctive materials have been introduced in clinical practice, including permanent synthetic meshes and absorbable meshes. The latter, such as polyglactin (Vicryl™) and polydioxanone (PDO), provide temporary mechanical support during the early phases of wound healing and are subsequently fully resorbed, thereby avoiding long-term foreign body presence [4,5].

In addition, implant-based breast reconstruction without mesh remains a viable option in carefully selected patients. However, current evidence remains heterogeneous, and large-scale randomized controlled trials are lacking. Consequently, it is still unclear whether absorbable meshes provide a significant long-term advantage compared with mesh-free reconstruction or reconstruction using acellular dermal matrix (ADM).

Within the expanding biomaterial landscape, absorbable synthetic meshes represent a distinct category of temporary structural scaffolds. Unlike emerging injectable or flowable matrices, which are designed primarily for soft-tissue augmentation or regenerative applications, absorbable sheet-form meshes provide temporary mechanical support during the early postoperative period. Their clinical relevance may therefore be particularly important in selected immediate implant-based reconstructions in which temporary implant stabilization, avoidance of permanent synthetic material, and reduced material costs are desired.

Therefore, the aim of this systematic review was to evaluate the outcomes of implant-based breast reconstruction following subcutaneous mastectomy using absorbable synthetic meshes and to compare them with mesh-free reconstruction and acellular dermal matrix (ADM)-assisted reconstruction, focusing on surgical, aesthetic, quality-of-life, and economic outcomes.

## 2. Materials and Methods

### 2.1. Literature Search Strategy

**Figure 1:**
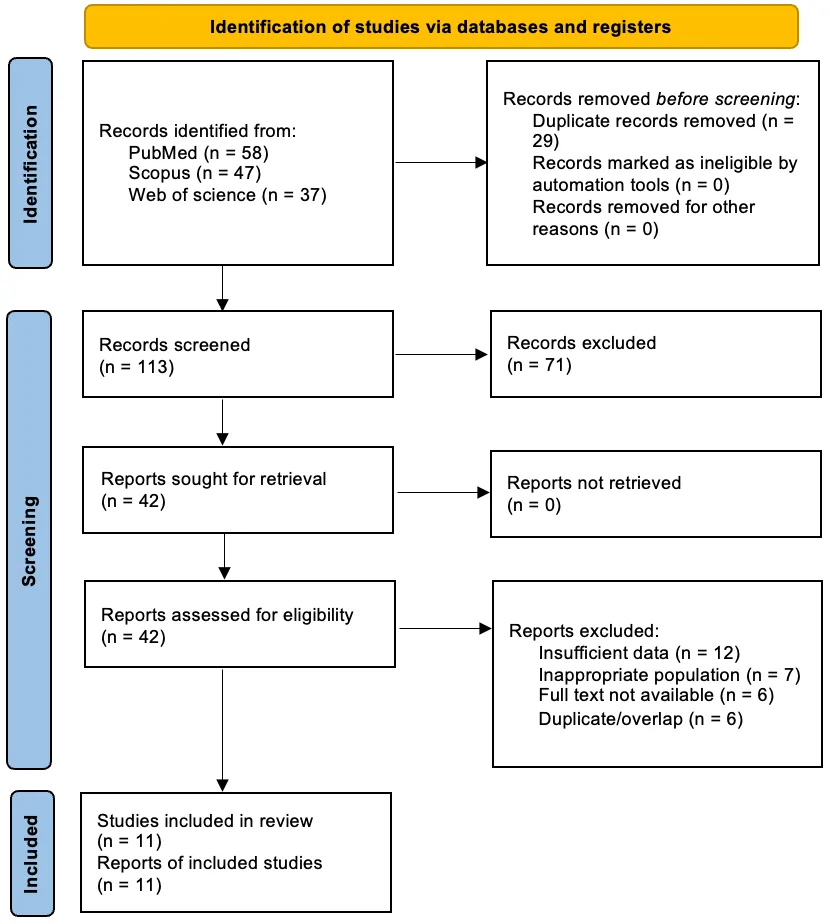
PRISMA 2020 flow diagram illustrating the study selection process.

This systematic review was conducted and reported in accordance with the Preferred Reporting Items for Systematic Reviews and Meta-Analyses (PRISMA 2020) statement [6]. The PRISMA 2020 flow diagram is presented in Figure 1, and the completed PRISMA 2020 checklist is provided as Supplementary Material. This review was not prospectively registered.

A comprehensive search of the literature was performed using PubMed, Scopus, and Web of Science databases from inception to July 2025. Only studies published in English were included.

The following keywords and their combinations were used: “polyglactin mesh,” “Vicryl mesh,” “polydioxanone mesh,” “PDO mesh,” “prepectoral implant breast reconstruction,” “subpectoral reconstruction,” “immediate breast reconstruction,” and “mesh-free reconstruction.”

In addition, the reference lists of relevant studies were manually reviewed. Recent issues of leading journals in the field, including *Plastic and Reconstructive Surgery*, *Journal of Plastic, Reconstructive & Aesthetic Surgery*, *Annals of Plastic Surgery*, and *Breast Cancer Research and Treatment*, were also screened to identify additional eligible studies.

### 2.2. Study Selection

The initial search identified 142 studies (PubMed n = 58, Scopus n = 47, Web of Science n = 37). After removal of duplicates (n = 29), 113 studies remained for screening based on titles and abstracts.

During the screening process, 71 studies were excluded due to irrelevance to the research topic or failure to meet the inclusion criteria. A total of 42 full-text articles were assessed for eligibility, of which 31 were excluded due to insufficient data, inappropriate study populations, or methodological limitations.

Ultimately, 11 studies were included in the final qualitative analysis.

### 2.3. Inclusion Criteria

Studies were included if they met the following criteria:

1. Prospective and retrospective clinical studies evaluating implant-based breast reconstruction using absorbable synthetic meshes, acellular dermal matrix (ADM), or mesh-free reconstruction were eligible for inclusion.
2. Patients undergoing subcutaneous mastectomy followed by immediate unilateral or bilateral implant-based breast reconstruction.
3. Explicit use of absorbable polyglactin or polydioxanone mesh, or comparison with mesh-free reconstruction or reconstruction using acellular dermal matrix (ADM).
4. Reported clinical outcomes, including rates of infection, seroma, skin necrosis, capsular contracture, implant loss, or reoperation, as well as aesthetic outcomes and quality-of-life measures.

### 2.4. Exclusion Criteria

Studies were excluded if they met any of the following criteria:

1. Animal or in vitro studies.
2. Studies focusing exclusively on aesthetic breast augmentation unrelated to oncologic reconstruction, except those describing material properties.
3. Studies without full-text availability or those providing insufficient outcome data.

### 2.5. Data Extraction and Analysis

Data were extracted from each included study, including publication year, authors, study design, number of patients, type of reconstruction (subpectoral or prepectoral), type of mesh used, complication rates (including implant loss, reoperation, capsular contracture), aesthetic outcomes, patient-reported quality-of-life measures, and economic data.

Two reviewers independently performed data extraction to ensure consistency and reduce bias.

Due to the heterogeneity of included studies in terms of surgical techniques, patient selection, outcome measures, and follow-up duration, quantitative meta-analysis was not feasible. Therefore, a narrative synthesis was performed to summarize clinical, aesthetic, and economic outcomes.

## 3. Results

### 3.1. Material Characteristics and Resorption Profiles

Polyglactin (Vicryl™, PG910) is a braided copolymer composed of glycolide and lactide, providing mechanical support for approximately 2–3 weeks and gradually losing tensile strength over 4–6 weeks, with complete resorption occurring within 8–12 weeks [4,5]. Polydioxanone (PDO) is a monofilament material with a longer resorption time of approximately 6–7 months, allowing prolonged implant stabilization, particularly in patients with thinner, soft tissue coverage [4,5]. Both materials are fully absorbable and are associated with a reduced risk of long-term foreign body reaction compared with non-absorbable meshes.

Eleven primary clinical studies evaluating implant-based breast reconstruction using absorbable synthetic meshes, acellular dermal matrix (ADM), or mesh-free techniques were included in the clinical outcomes analysis. Together, these studies comprised 549 patients (698 breasts). The characteristics of the included studies are summarized in Table 1.

The postoperative clinical outcomes reported in the included studies are summarized in Table 2. Owing to differences in study design, patient characteristics, and outcome definitions, complication rates are presented as reported in the original publications.

### 3.2. Risk-of-Bias Assessment

The methodological quality of the included clinical studies was variable (Table 3). Comparative cohort studies generally demonstrated adequate cohort selection and outcome ascertainment; however, residual confounding remained a concern because adjustment for differences in patient characteristics and treatment-related factors was limited. Most of the remaining studies were single-arm observational case series and were generally adequate in terms of participant selection and outcome reporting, but their methodological quality was limited by the absence of comparator groups, selective patient populations, small sample sizes, and, in some studies, relatively short follow-up. Overall, most studies were considered to have a moderate risk of bias, with no study providing high-certainty comparative evidence.

### 3.3. Clinical Outcomes with Polyglactin Mesh

Across the included primary clinical studies, postoperative complication rates following implant-based breast reconstruction using polyglactin (Vicryl) mesh were generally low, although substantial variation was observed between studies. Reported infection rates ranged from 0% to 8.7%, hematoma from 0% to 7.0%, seroma from 0% to 3.1%, implant failure from 0% to 6.3%, wound dehiscence from 0% to 8.0%, flap necrosis from 0% to 6.3%, implant malposition from 0% to 8.9%, and capsular contracture from 0% to 10.7%. No cases of red breast syndrome were reported, while implant rippling and implant rupture were rarely observed.

### 3.4. Clinical Outcomes with Polydioxanone Mesh

Clinical evidence regarding polydioxanone (PDO) mesh remains limited. In the included study, postoperative outcomes were favorable, with an infection rate of 7.1% and no reported cases of hematoma, seroma, implant failure, wound dehiscence, flap necrosis, red breast syndrome, implant malposition, capsular contracture, implant rippling, or implant rupture [10].

Evidence regarding polydioxanone mesh was limited to a single study involving 7 patients and 14 reconstructed breasts. Although the reported short-term outcomes were favorable, the small sample size and absence of comparative data preclude conclusions regarding its safety, effectiveness, or equivalence to polyglactin mesh or ADM.

### 3.5. Clinical Outcomes with Acellular Dermal Matrix

Reconstruction using acellular dermal matrix (ADM) indicates infection rates ranging from 0% to 3.6%, hematoma from 3.3% to 5.5%, seroma from 5.0% to 12.7%, implant failure from 0% to 3.3%, wound dehiscence from 1.6% to 10.9%, and flap necrosis from 1.6% to 3.6%. Red breast syndrome was reported in up to 1.6% of cases, whereas no cases of implant malposition, capsular contracture, implant rippling, or implant rupture were reported in the included studies [9,11].

### 3.6. Mesh-Free Reconstruction Outcomes

Mesh-free reconstruction demonstrated infection rates ranging from 0% to 2.4%, hematoma from 0% to 1.6%, seroma from 0% to 5.0%, implant failure from 1.2% to 8.7%, wound dehiscence from 0% to 2.2%, flap necrosis from 0% to 1.6%, implant malposition from 0% to 21.7%, capsular contracture from 0% to 10.6%, and implant rupture from 0% to 5.9%. No cases of red breast syndrome or implant rippling were reported [7,11,17].

### 3.7. Economic Outcomes

Only one comparative clinical study directly evaluated the economic impact of absorbable mesh and ADM. Mookerjee et al. reported a substantial reduction in material costs when Vicryl mesh was used compared with ADM (USD 760 vs. USD 9033 per reconstruction), together with a shorter operative time [9].

The use of Vicryl mesh instead of acellular dermal matrices resulted in direct material cost savings of $172,112 in 10 months. From these data, direct-to-implant reconstruction with Vicryl absorbable mesh costs less than two-thirds of the total expenditures of the previous most cost-efficient technique [12].

## 4. Discussion

This systematic review suggests that absorbable synthetic meshes, predominantly polyglactin mesh, may represent a feasible and potentially cost-saving option for selected implant-based breast reconstructions. Evidence regarding PDO remains insufficient for firm conclusions. The available evidence suggests that absorbable meshes are associated with clinical outcomes comparable to those reported for acellular dermal matrix (ADM) and mesh-free reconstruction. However, these findings should be interpreted with caution because the available evidence is predominantly derived from retrospective and single-arm observational studies, with substantial methodological and clinical heterogeneity and an overall moderate risk of bias [7,8,9,10,11,12,13,14,15,16,17].

### 4.1. Complication Profile

Across the included studies, polyglactin mesh was associated with relatively low rates of postoperative complications. Seroma formation ranged from approximately 1% to 5% and was typically self-limiting, rarely requiring surgical intervention [7,8,9]. Reported rates of skin necrosis (4–8%) and infection (2–5%) were generally comparable to, or in some studies slightly lower than, those observed following ADM-assisted or mesh-free reconstruction [7,18]. In addition, some comparative studies reported lower reconstruction failure rates with polyglactin mesh than with mesh-free techniques (e.g., 6.3% vs. 8.7%) [8,18]. These findings suggest that polyglactin mesh may provide adequate short-term implant support during the early postoperative healing period; however, they should be interpreted cautiously because of the limited number of comparative studies, heterogeneity of the available evidence, and identified risk of bias.

The range of biomaterials available for implant-based breast reconstruction continues to expand and includes multiple human-derived ADM products as well as newer injectable and flowable matrices. These materials should not be regarded as clinically interchangeable. Human-derived ADMs provide a biologic scaffold for tissue integration, whereas injectable and flowable matrices may be used primarily for soft-tissue reinforcement, contour correction, or regenerative purposes. In contrast, absorbable sheet-form synthetic meshes are intended to provide temporary structural support to the implant during early healing. Accordingly, the clinical relevance of absorbable synthetic meshes may lie in selected immediate implant-based reconstructions in which temporary stabilization and lower material costs are prioritized. Current evidence, however, is insufficient to establish their superiority over ADM or newer biomaterial technologies.

### 4.2. Aesthetic and Functional Outcomes

Several studies have reported favorable aesthetic outcomes and high patient satisfaction following implant-based breast reconstruction with absorbable meshes [8,9]. However, these findings should be interpreted with caution because aesthetic outcomes were assessed using different methodologies and were not consistently reported across studies.

### 4.3. Economic Considerations

One potential advantage of absorbable meshes is their lower material cost compared with ADM. Mookerjee et al. reported that the use of polyglactin mesh significantly reduced operative time—nearly by half (35.7 vs. 68 min with ADM)—and decreased material costs by up to 91%, corresponding to savings of approximately USD 8273 per breast [9].

Although lower rates of major complications and implant loss could theoretically reduce overall treatment costs by decreasing the need for reoperations, prolonged hospitalization, and additional surgical materials, these potential downstream savings have not been formally evaluated and should therefore be interpreted cautiously [7,10].

### 4.4. Comparison with Other Reconstruction Techniques

Large-scale analyses outside the studies included in this review, including the iBRA study (Potter et al.) and subsequent evaluations by DeLong et al., have reported no significant differences in early postoperative complication rates within the first three months between biosynthetic meshes, acellular dermal matrix (ADM), and mesh-free reconstruction [3,19]. Similarly, Arnautovic et al. reported comparable outcomes across different reconstructive approaches [20]. Taken together, these findings suggest that the choice of implant support material should consider not only complication rates but also patient characteristics, surgeon experience, material availability, and economic factors.

### 4.5. Patient Selection and Risk Factors

The study by Vohra et al. reported a higher risk of postoperative complications among patients with elevated body mass index and comorbidities such as diabetes and hypertension, emphasizing the importance of careful patient selection [8]. These findings suggest that individualized treatment planning remains important regardless of the reconstruction technique used.

### 4.6. Limitations

Despite promising results, the current evidence base remains limited. Most included studies were retrospective, with relatively small sample sizes and short follow-up periods, typically not exceeding 12 months. Consequently, long-term outcomes, including capsular contracture, implant malposition, and durability of aesthetic results, remain insufficiently evaluated.

Furthermore, the lack of standardized outcome measures, particularly for quality of life and patient satisfaction, limits the ability to perform direct comparisons between studies.

The comparison between absorbable meshes and ADM should be interpreted cautiously. Most available evidence originates from retrospective observational studies with heterogeneous patient populations, surgical techniques, indications for mesh use, and follow-up periods. Therefore, direct comparison between different materials is subject to considerable selection bias and confounding.

Another important limitation is the marked heterogeneity of the available clinical evidence. The included clinical studies evaluated different reconstructive materials (polyglactin mesh, polydioxanone mesh, ADM, and mesh-free reconstruction), different surgical techniques (prepectoral and subpectoral reconstruction), and different study designs, including retrospective and prospective studies. This heterogeneity limited direct comparisons between studies and precluded quantitative meta-analysis. Therefore, the findings should be interpreted as a qualitative synthesis of the currently available evidence rather than definitive comparative evidence.

The formal risk-of-bias assessment further highlighted important methodological limitations of the available evidence. Most included studies were judged to have a moderate risk of bias, primarily because of their retrospective or single-arm observational designs, limited adjustment for potential confounding, selective patient populations, small sample sizes, and relatively short follow-up. These limitations reduce the certainty of direct comparisons between reconstructive materials and should be considered when interpreting the findings of this review.

In addition, the economic evidence was limited to a small number of studies reporting primarily material costs and operative time. No formal cost-effectiveness analyses were identified; therefore, the present review cannot establish the cost-effectiveness of absorbable meshes relative to ADM or mesh-free reconstruction.

The review process itself also has limitations. Only English-language studies were included, and grey literature was not systematically searched, which may have influenced the completeness of the available evidence.

### 4.7. Future Directions

Future research should focus on well-designed prospective, multicenter studies with larger patient cohorts and longer follow-up periods. Standardized outcome measures, including validated quality-of-life instruments, are essential to better evaluate both clinical and patient-reported outcomes.

### 4.8. Summary

Overall, the findings of this review suggest that absorbable synthetic meshes—particularly polyglactin mesh—may represent a feasible option for selected implant-based breast reconstructions, with clinical outcomes generally comparable to those reported for ADM-assisted or mesh-free reconstruction. Available evidence also suggests substantially lower material costs and shorter operative times compared with ADM; however, these findings should not be interpreted as evidence of formal cost-effectiveness. Given the predominantly observational evidence, methodological heterogeneity, and identified risk of bias, further prospective comparative studies and dedicated health-economic evaluations are required before definitive conclusions can be drawn.

## 5. Conclusions

The available evidence suggests that polyglactin mesh may represent a feasible option for temporary implant support in selected patients undergoing immediate implant-based breast reconstruction, with potential advantages in terms of material costs and operative time. However, the evidence remains predominantly retrospective and clinically heterogeneous, and the identified risk of bias limits the certainty of these findings. Evidence regarding polydioxanone mesh is limited to a single small study and is currently insufficient to establish its comparative safety, effectiveness, or economic value.

Polyglactin mesh may offer an economic advantage, with reported reductions in material costs of up to 90% and shorter operative times compared with acellular dermal matrix (ADM). However, these findings reflect material cost savings and operative efficiency rather than formal cost-effectiveness. Potential downstream cost savings related to complications, reoperations, or hospitalization have not been formally evaluated.

The most frequently reported complications—including seroma, skin necrosis, infection, and hematoma—were generally infrequent; however, interpretation of these findings is limited by study heterogeneity and risk of bias. Patient selection remains an important factor influencing outcomes, with higher body mass index and comorbidities such as diabetes and hypertension associated with an increased risk of postoperative complications.

Several studies reported favorable aesthetic outcomes and high levels of patient satisfaction; however, the lack of standardized assessment methods and limited long-term follow-up restricts objective comparison across studies.

Future research should focus on well-designed prospective, multicenter studies with larger patient cohorts and longer follow-up periods to better evaluate long-term clinical outcomes, cost-effectiveness, and patient-reported quality of life.

## Figures and Tables

**Table 1:** Characteristics of the included primary clinical studies.

Study	Plane	Mesh Type	Patients	Breasts	Follow-Up
Ganz et al., 2015 [7]	Submuscular and partial submuscular	No mesh	40	46	3 months
Ganz et al., 2015 [7]	Submuscular and partial submuscular	Vicryl (Polyglactin 910)	97	112	3 months
Vohra et al., 2024 [8]	Prepectoral	Vicryl (Polyglactin 910)	28	32	12 months
Mookerjee et al., 2023 [9]	Prepectoral	Vicryl (Polyglactin 910)	12	23	3 months
Mookerjee et al., 2023 [9]	Prepectoral	ADM	34	55	3 months
Qui et al., 2022 [10]	Prepectoral	PDO (Polydioxanone)	7	14	9 months
Hajiesmaeili et al., 2024 [11]	Prepectoral	No mesh	49	60	6 months
Hajiesmaeili et al., 2024 [11]	Prepectoral	ADM	52	60	6 months
Tessler et al., 2014 [12]	Submuscular	Vicryl (Polyglactin 910)	50	76	1,2 years
Haynes et al., 2014 [13]	Prepectoral	Vicryl (Polyglactin 910)	38	46	3,6 years
Gunnarsson et al., 2018 [14]	Prepectoral	Vicryl (Polyglactin 910)	27	47	11 months
Hashimoto et al., 2017 [15]	Prepectoral	Vicryl (Polyglactin 910)	80	81	NR
Kobraei et al., 2016 [16]	Prepectoral	Vicryl (Polyglactin 910)	13	23	NR
Engel et al., 2013 [17]	Submuscular	No mesh	22	23	42 months

**Table 2:** Postoperative clinical outcomes of the included primary clinical studies.

Study	Mesh Type	Overall	Infection	Hematoma	Seroma	Implant Failure	Wound Dehiscence	Necrosis	RBS	Malposition	Contracture
Ganz et al., 2015 [7]	No mesh	19 (41.3%)	1 (2.2%)	0	0	4 (8.7%)	1 (2.2%)	0	0	10 (21.7%)	3 (6.5%)
Ganz et al., 2015 [7]	Vicryl	38 (33.9%)	3 (2.7%)	1 (0.9%)	0	7 (6.3%)	9 (8.0%)	0	0	10 (8.9%)	12 (10.7%)
Vohra et al., 2024 [8]	Vicryl	4 (12.5%)	0	0	1 (3.1%)	1 (3.1%)	1 (3.1%)	1 (3.1%)	0	0	0
Mookerjee et al., 2023 [9]	Vicryl	4 (17.4%)	2 (8.7%)	1 (4.3%)	0	0	0	1 (4.3%)	0	0	0
Mookerjee et al., 2023 [9]	ADM	20 (36.4%)	2 (3.6%)	3 (5.5%)	7 (12.7%)	0	6 (10.9%)	2 (3.6%)	0	0	0
Qui et al., 2022 [10]	PDO	1 (7.1%)	1 (7.1%)	0	0	0	0	0	0	0	0
Hajiesmaeili et al., 2024 [11]	No mesh	8 (13.3%)	0	1 (1.6%)	3 (5%)	2 (3.3%)	1 (1.6%)	1 (1.6%)	0	0	0
Hajiesmaeili et al., 2024 [11]	ADM	9 (15%)	0	2 (3.3%)	3 (5%)	2 (3.3%)	1 (1.6%)	1 (1.6%)	1 (1.6%)	0	0
Tessler et al., 2014 [12]	Vicryl	5 (6.6%)	1 (1.3%)	0	0	1 (1.3%)	0	2 (2.6%)	0	0	1 (1.3%)
Haynes et al., 2014 [13]	Vicryl	7 (15.2%)	3 (6.5%)	0	1 (2.2%)	1 (2.2%)	0	2 (4.3%)	0	0	0
Gunnarsson et al., 2018 [14]	Vicryl	5 (10.6%)	1 (2.1%)	2 (4.2%)	0	0	1 (2.1%)	1 (2.1%)	0	0	0
Hashimoto et al., 2017 [15]	Vicryl	8 (10.0%)	3 (3.75%)	0	0	0	0	5 (6.25%)	0	0	0
Kobraei et al., 2016 [16]	Vicryl	5 (38.6%)	1 (7%)	1 (7%)	3 (23%)	0	0	0	0	0	0
Engel et al., 2013 [17]	No mesh	18 (21.2%)	2 (2.4%)	1 (1.2%)	0	1 (1.2%)	0	0	0	0	9 (10.6%)

**Table 3:** Risk-of-bias and methodological quality assessment of the included clinical studies.

Study	Study Design	Assessment Tool	Selection	Comparability/Confounding	Outcome Assessment/Follow-Up	Overall Appraisal
Ganz et al., 2015 [7]	Retrospective comparative cohort	NOS	Good	Moderate	Good	**Moderate risk of bias**
Vohra et al., 2024 [8]	Retrospective single-arm case series	JBI Case Series	Good	N/A	Good	**Moderate risk of bias**
Mookerjee et al., 2023 [9]	Retrospective comparative cohort	NOS	Good	Moderate–poor	Moderate	**Moderate risk of bias**
Qiu et al., 2022 [10]	Prospective consecutive single-arm case series	JBI Case Series	Good	N/A	Good	**Low–moderate risk of bias**
Hajiesmaeili et al., 2024 [11]	Retrospective comparative cohort	NOS	Good	Moderate–poor	Good	**Moderate risk of bias**
Tessler et al., 2014 [12]	Retrospective consecutive case series	JBI Case Series	Good	N/A	Good	**Moderate risk of bias**
Haynes & Kreithen, 2014 [13]	Observational case series based on a prospective database	JBI Case Series	Good	N/A	Good	**Moderate risk of bias**
Gunnarsson et al., 2018 [14]	Prospective case series	JBI Case Series	Moderate	N/A	Good	**Moderate risk of bias**
Hashimoto et al., 2017 [15]	Retrospective case series	JBI Case Series	Moderate	N/A	Good	**Moderate risk of bias**
Kobraei et al., 2016 [16]	Retrospective case series	JBI Case Series	Moderate	N/A	Moderate	**Moderate–high risk of bias**
Engel et al., 2013 [17]	Consecutive observational case series	JBI Case Series	Good	N/A	Good	**Moderate risk of bias**

Abbreviations: NOS, Newcastle–Ottawa Scale; JBI, Joanna Briggs Institute; N/A, not applicable. Overall appraisal reflects a qualitative judgment based on the applicable domains rather than a numerical cut-off score.

## Data Availability

No new data were created or analyzed in this study. Data sharing is not applicable to this article.

## Supplementary Materials

The following supporting information can be downloaded at: https://www.mdpi.com/article/10.3390/medicina62091682/s1, PRISMA 2020 Checklist.

## Abbreviations

The following abbreviations are used in this manuscript:

ADM	Acellular dermal matrix
PDO	Polydioxanone
RBS	Red breast syndrome

## References

[1] Nolan I.T., Farajzadeh M.M., Bekisz J.M.M., Boyd C.J.M., Gibson E.G., Salibian A.A. (2024). Long-term outcomes in breast reconstruction with synthetic absorbable mesh. Plast. Reconstr. Surg. Glob. Open.

[2] Faulkner H.R., Shikowitz-Behr L., McLeod M., Wright E., Hulsen J., Austen W.G. (2020). The use of absorbable mesh in implant-based breast reconstruction: A 7-year review. Plast. Reconstr. Surg..

[3] Potter S., Conroy E.J., Cutress R.I., Williamson P.R., Whisker L., Thrush S., Skillman J., Barnes N.L.P., Mylvaganam S., Teasdale E. (2019). Short-term safety outcomes of mastectomy and immediate implant-based breast reconstruction with and without mesh (iBRA): A multicentre, prospective cohort study. Lancet Oncol..

[4] Klinge U., Schumpelick V., Klosterhalfen B. (2001). Functional assessment and tissue response of short- and long-term absorbable surgical meshes. Biomaterials.

[5] Heidari B.S., Dodda J.M., El-Khordagui L.K., Focarete M.L., Maroti P., Toth L., Pacilio S., El-Habashy S.E., Boateng J., Catanzano O. (2024). Emerging materials and technologies for advancing bioresorbable surgical meshes. Acta Biomater..

[6] Page M.J., McKenzie J.E., Bossuyt P.M., Boutron I., Hoffmann T.C., Mulrow C.D., Shamseer L., Tetzlaff J.M., Akl E.A., Brennan S.E. (2021). The PRISMA 2020 statement: An updated guideline for reporting systematic reviews. BMJ.

[7] Ganz O.M., Tobalem M., Perneger T., Lam T., Modarressi A., Elias B., Pittet B. (2015). Risks and benefits of using an absorbable mesh in one-stage immediate breast reconstruction: A comparative study. Plast. Reconstr. Surg..

[8] Vohra L.M., Virji S.N., Arain H., Abedin I., Zeeshan S. (2024). Early post-operative outcome of pre-pectoral implant-based immediate total breast reconstruction with polyglactin 910 (Vicryl™) mesh. BMC Surg..

[9] Mookerjee V.G., Stögner V.A., Alper D.P., Evans B.G.A., Pomahac B. (2023). Single stitch Vicryl mesh wrap for prepectoral implant breast reconstruction. Plast. Reconstr. Surg. Glob. Open.

[10] Qiu C.S., Seth A.K. (2022). Early clinical outcomes of polydioxanone mesh for prepectoral prosthetic breast reconstruction. Plast. Reconstr. Surg. Glob. Open.

[11] Hajiesmaeili H., Shirazi S., Agrawal K., Vidya R. (2024). A comparative study of one-stage pre-pectoral implant breast reconstruction with and without mesh. Cureus.

[12] Tessler O., Austen W., Smith B., Maman D., Reish R. (2014). Beyond Biologics: Absorbable Mesh as a Low-Cost, Low-Complication Sling for Implant-Based Breast Reconstruction. Plast. Reconstr. Surg..

[13] Haynes D.F., Kreithen J.C. (2014). Vicryl mesh in expander/implant breast reconstruction: Long-term follow-up in 38 patients. Plast. Reconstr. Surg..

[14] Gunnarsson G.L., Thomsen J.B. (2018). Prepectoral Hammock and Direct-to-implant Breast Reconstruction in 10 Minutes: A Focus on Technique. Plast. Reconstr. Surg. Glob. Open.

[15] Hashimoto Y., Watanabe N., Yuasa T., Suzuki Y., Saisho H. (2017). Breast reconstruction with absorbable mesh sling: Dynamic infrared thermography of skin envelope. Gland. Surg..

[16] Kobraei E.M., Cauley R., Gadd M., Austen W.G., Liao E.C. (2016). Avoiding Breast Animation Deformity with Pectoralis-Sparing Subcutaneous Direct-to-Implant Breast Reconstruction. Plast. Reconstr. Surg. Glob. Open.

[17] Engel H., Huang J.J., Lin C.Y., Lam W.L., Gazyakan E., Cheng M.H. (2013). Subcutaneous tissue expansion and subsequent subpectoral implantation for breast reconstruction in Asian patients: Safety and outcome. Ann. Plast. Surg..

[18] Rodriguez-Unda N., Leiva S., Cheng H.T., Seal S.M., Cooney C.M., Rosson G.D. (2015). Low incidence of complications using polyglactin 910 mesh in breast reconstruction: A systematic review. J. Plast. Reconstr. Aesthetic Surg..

[19] DeLong M.R., Tandon V.J., Bertrand A.A.M., MacEachern M.M., Goldberg M., Salibian A., Pusic A.L.M., Festekjian J.H., Wilkins E.G. (2021). Review of outcomes in prepectoral prosthetic breast reconstruction with and without surgical mesh assistance. Plast. Reconstr. Surg..

[20] Arnautovic A., Williams S., Ash M., Menon A., Shauly O., Losken A. (2025). Outcomes in implant-based breast reconstruction utilizing biosynthetic mesh: A meta-analysis. Aesthetic Surg. J..

