# Supplementary material for: Absorbable Synthetic Meshes in Implant-Based Breast Reconstruction After Subcutaneous Mastectomy: A Systematic Review with Clinical and Economic Considerations
Source: Medicina (Kaunas). 2026 Sep 2;62(9):1682. doi: 10.3390/medicina62091682 (PMC13608941; doi:10.3390/medicina62091682)
Supplement: Supplementary file 1 [file medicina-62-01682-s001.zip › medicina-4427518-supplementary.pdf]

PRISMA 2020 Checklist

Section and Topic	Item #	Checklist item	Location where item is reported
**TITLE**			
Title	1	Identify the report as a systematic review.	Title page
**ABSTRACT**			
Abstract	2	See the PRISMA 2020 for Abstracts checklist.	Abstract
**INTRODUCTION**			
Rationale	3	Describe the rationale for the review in the context of existing knowledge.	Introduction (pp. 2-3)
Objectives	4	Provide an explicit statement of the objective(s) or question(s) the review addresses.	Introduction (end of Introduction, p.3)
**METHODS**			
Eligibility criteria	5	Specify the inclusion and exclusion criteria for the review and how studies were grouped for the syntheses.	Methods – Inclusion and Exclusion Criteria (p. 5)
Information sources	6	Specify all databases, registers, websites, organisations, reference lists and other sources searched or consulted to identify studies. Specify the date when each source was last searched or consulted.	Methods – Literature Search Strategy (pp. 4-5)
Search strategy	7	Present the full search strategies for all databases, registers and websites, including any filters and limits used.	Literature Search Strategy (p. 4)
Selection process	8	Specify the methods used to decide whether a study met the inclusion criteria of the review, including how many reviewers screened each record and each report retrieved, whether they worked independently, and if applicable, details of automation tools used in the process.	Methods – Study Selection (p. 5)
Data collection process	9	Specify the methods used to collect data from reports, including how many reviewers collected data from each report, whether they worked independently, any processes for obtaining or confirming data from study investigators, and if applicable, details of automation tools used in the process.	Methods – Data Extraction and Analysis (p. 5)
Data items	10a	List and define all outcomes for which data were sought. Specify whether all results that were compatible with each outcome domain in each study were sought (e.g. for all measures, time points, analyses), and if not, the methods used to decide which results to collect.	Methods – Data Extraction and Analysis (p. 5)
	10b	List and define all other variables for which data were sought (e.g. participant and intervention characteristics, funding sources). Describe any assumptions made about any missing or unclear information.	Methods – Data Extraction and Analysis (p. 5)
Study risk of bias assessment	11	Specify the methods used to assess risk of bias in the included studies, including details of the tool(s) used, how many reviewers assessed each study and whether they worked independently, and if applicable, details of automation tools used in the process.	Methods – Data

PRISMA 2020 Checklist

Section and Topic	Item #	Checklist item	Location where item is reported
			Extraction and Analysis (p. 5)
Effect measures	12	Specify for each outcome the effect measure(s) (e.g. risk ratio, mean difference) used in the synthesis or presentation of results.	Not applicable (no meta-analysis performed)
Synthesis methods	13a	Describe the processes used to decide which studies were eligible for each synthesis (e.g. tabulating the study intervention characteristics and comparing against the planned groups for each synthesis (item #5)).	Methods – Study Selection (p. 5)
	13b	Describe any methods required to prepare the data for presentation or synthesis, such as handling of missing summary statistics, or data conversions.	Not aplicable
	13c	Describe any methods used to tabulate or visually display results of individual studies and syntheses.	Results – Tables 1-2 (pp. 7-8)
	13d	Describe any methods used to synthesize results and provide a rationale for the choice(s). If meta-analysis was performed, describe the model(s), method(s) to identify the presence and extent of statistical heterogeneity, and software package(s) used.	Methods – Data Extraction and Analysis (narrative synthesis, p. 5)
	13e	Describe any methods used to explore possible causes of heterogeneity among study results (e.g. subgroup analysis, meta-regression).	Not applicable.
	13f	Describe any sensitivity analyses conducted to assess robustness of the synthesized results.	Not applicable.
Reporting bias assessment	14	Describe any methods used to assess risk of bias due to missing results in a synthesis (arising from reporting biases).	Not performed
Certainty assessment	15	Describe any methods used to assess certainty (or confidence) in the body of evidence for an outcome.	Not performed
**RESULTS**			
Study selection	16a	Describe the results of the search and selection process, from the number of records identified in the search to the number of studies included in the review, ideally using a flow diagram.	Results – Study Selection (pp. 6-7), Figure 1
	16b	Cite studies that might appear to meet the inclusion criteria, but which were excluded, and explain why they were excluded.	Results – Study selection (p. 6), Figure 1
Study characteristics	17	Cite each included study and present its characteristics.	Results – Tables 1-2 (pp. 7-8)
Risk of bias in studies	18	Present assessments of risk of bias for each included study.	Not applicable. No formal risk-

Section and Topic	Item #	Checklist item	Location where item is reported
			of-bias assessment was performed because of substantial methodological heterogeneity
Results of individual studies	19	For all outcomes, present, for each study: (a) summary statistics for each group (where appropriate) and (b) an effect estimate and its precision (e.g. confidence/credible interval), ideally using structured tables or plots.	Results – Tables 1-2 (pp. 7-8)
Results of syntheses	20a	For each synthesis, briefly summarise the characteristics and risk of bias among contributing studies.	Results – Tables 1–2 and narrative synthesis (pp. 7–9). No formal risk-of-bias assessment was performed.
	20b	Present results of all statistical syntheses conducted. If meta-analysis was done, present for each the summary estimate and its precision (e.g. confidence/credible interval) and measures of statistical heterogeneity. If comparing groups, describe the direction of the effect.	Not applicable (no meta-analysis performed)
	20c	Present results of all investigations of possible causes of heterogeneity among study results.	Results (heterogeneity discussed, pp. 7-8)
	20d	Present results of all sensitivity analyses conducted to assess the robustness of the synthesized results.	Not applicable
Reporting biases	21	Present assessments of risk of bias due to missing results (arising from reporting biases) for each synthesis assessed.	Not performed
Certainty of evidence	22	Present assessments of certainty (or confidence) in the body of evidence for each outcome assessed.	Not performed
**DISCUSSION**			
Discussion	23a	Provide a general interpretation of the results in the context of other evidence.	Discussion (pp. 8-9)
	23b	Discuss any limitations of the evidence included in the review.	Discussion – Limitations (p. 9)

Section and Topic	Item #	Checklist item	Location where item is reported
	23c	Discuss any limitations of the review processes used.	The review process itself also has several limitations. Only English-language studies were included, unpublished literature was not systematically searched, and no formal risk-of-bias assessment was performed. These factors may have influenced the completeness and certainty of the synthesized evidence.
	23d	Discuss implications of the results for practice, policy, and future research.	Discussion / Conclusions (pp. 9-10)
**OTHER INFORMATION**			
Registration and protocol	24a	Provide registration information for the review, including register name and registration number, or state that the review was not registered.	Methods – Literature Search Strategy (p. 4): Review was not prospectively registered
	24b	Indicate where the review protocol can be accessed, or state that a protocol was not prepared.	No review protocol was prepared

PRISMA 2020 Checklist

Section and Topic	Item #	Checklist item	Location where item is reported
	24c	Describe and explain any amendments to information provided at registration or in the protocol.	Not applicable
Support	25	Describe sources of financial or non-financial support for the review, and the role of the funders or sponsors in the review.	Funding (p. 10)
Competing interests	26	Declare any competing interests of review authors.	Conflicts of interest (p. 10)
Availability of data, code and other materials	27	Report which of the following are publicly available and where they can be found: template data collection forms; data extracted from included studies; data used for all analyses; analytic code; any other materials used in the review.	Data Availability Statement (p. 10)

From: Page MJ, McKenzie JE, Bossuyt PM, Boutron I, Hoffmann TC, Mulrow CD, et al. The PRISMA 2020 statement: an updated guideline for reporting systematic reviews. BMJ 2021;372:n71. doi: 10.1136/bmj.n71. This work is licensed under CC BY 4.0. To view a copy of this license, visit https://creativecommons.org/licenses/by/4.0/
